# Mitochondrial phosphopantetheinylation is required for oxidative metabolism

**DOI:** 10.1016/j.metabol.2025.156413

**Published:** 2025-10-06

**Authors:** Pieter R. Norden, Riley J. Wedan, Samuel E.J. Preston, Morgan Canfield, Naomi Graber, Jacob Z. Longenecker, Olivia A. Pentecost, Elizabeth McLaughlin, Madeleine L. Hart, Sara M. Nowinski

**Affiliations:** a Department of Metabolism and Nutritional Programming, Van Andel Institute, Grand Rapids, MI, United States of America; b The Van Andel Institute Graduate School, Grand Rapids, MI, United States of America; c College of Human Medicine, Michigan State University, Grand Rapids, MI, United States of America

**Keywords:** Mitochondria, Fatty acid synthesis, Electron transport chain, Reductive carboxylation, Metabolism, Respiration, Phosphopantetheine

## Abstract

4′-Phosphopantetheinyl (4’PP) groups are essential co-factors added to target proteins by phosphopantetheinyl transferase (PPTase) enzymes. Although mitochondrial 4’PP-modified proteins have been described for decades, a mitochondrially-localized PPTase has never been found in mammals. We discovered that the cytoplasmic PPTase aminoadipate semialdehyde dehydrogenase phosphopantetheinyl transferase (AASDHPPT) is required for mitochondrial respiration and oxidative metabolism. Loss of AASDHPPT results in failed 4’PP modification of the mitochondrial acyl carrier protein and blunted activity of the mitochondrial fatty acid synthesis (mtFAS) pathway. We found that in addition to its cytoplasmic localization, AASDHPPT localizes to the mitochondrial matrix via an N-terminal mitochondrial targeting sequence contained within the first 20 amino acids of the protein. Our data show that this novel mitochondrial localization of AASDHPPT is required to support mtFAS activity and oxidative metabolism. We further identify five variants of uncertain significance in *AASDHPPT* that are likely pathogenic in humans due to loss of mtFAS activity.

## 1. Introduction

4′-phosphopantentheinylation is an essential post translational modification (PTM) whereby a 4′-phosphopantetheine (4’PP) group, derived from coenzyme A (CoA), is covalently attached to target proteins by a phosphopantetheinyltransferase (PPTase) enzyme [1]. Six mammalian proteins have been found to have a 4’PP-modified serine: three cytoplasmic proteins (Fatty Acid Synthase, FASN; Aminoadipate semialdehyde dehydrogenase, AASDH; and Aldehyde Dehydrogenase 1 Family Member L1, ALDH1L1), and three mitochondrial proteins (NADH:Ubiquinone Oxidoreductase Subunit AB1, NDUFAB1; Aldehyde Dehydrogenase 1 Family Member L2, ALDH1L2; and mitochondrial Dehydrogenase/Reductase 2, DHRS2) [1]. In mammalian genomes, there is only one annotated PPTase: aminoadipate-semialdehyde dehydrogenase-phosphopantetheinyl transferase (AASDHPPT), which was thought to exist solely in the cytoplasm [2]. This contrasts with other species such as *Arabidopsis thaliana* and *Saccharomyces cerevisiae,* which have dedicated mitochondrial PPTases, the activity of which cannot be compensated by cytoplasmic PPTases [3,4]. It was therefore unclear how 4’PP is attached to mitochondrial proteins in mammalian cells.

Since no mammalian mitochondrial PPTase had ever been described, we questioned whether an unidentified PPTase existed in mammals, or conversely if AASDHPPT (hereafter abbreviated PPT) was important for mitochondrial function regardless of its reported cytoplasmic localization. One prior study found that PPT could add a 4’PP group to purified mitochondrial proteins in vitro, but concluded that its activity was greater than 90 % cytoplasmic, raising questions as to whether this activity was biologically relevant [2]. We therefore set out to understand whether PPT is required for mitochondrial function and, if so, how it modifies mitochondrial targets despite existing in separate cellular compartments.

## 2. Results

### 2.1. AASDHPPT expression is required for oxidative mitochondrial metabolism

**Fig. 1.**
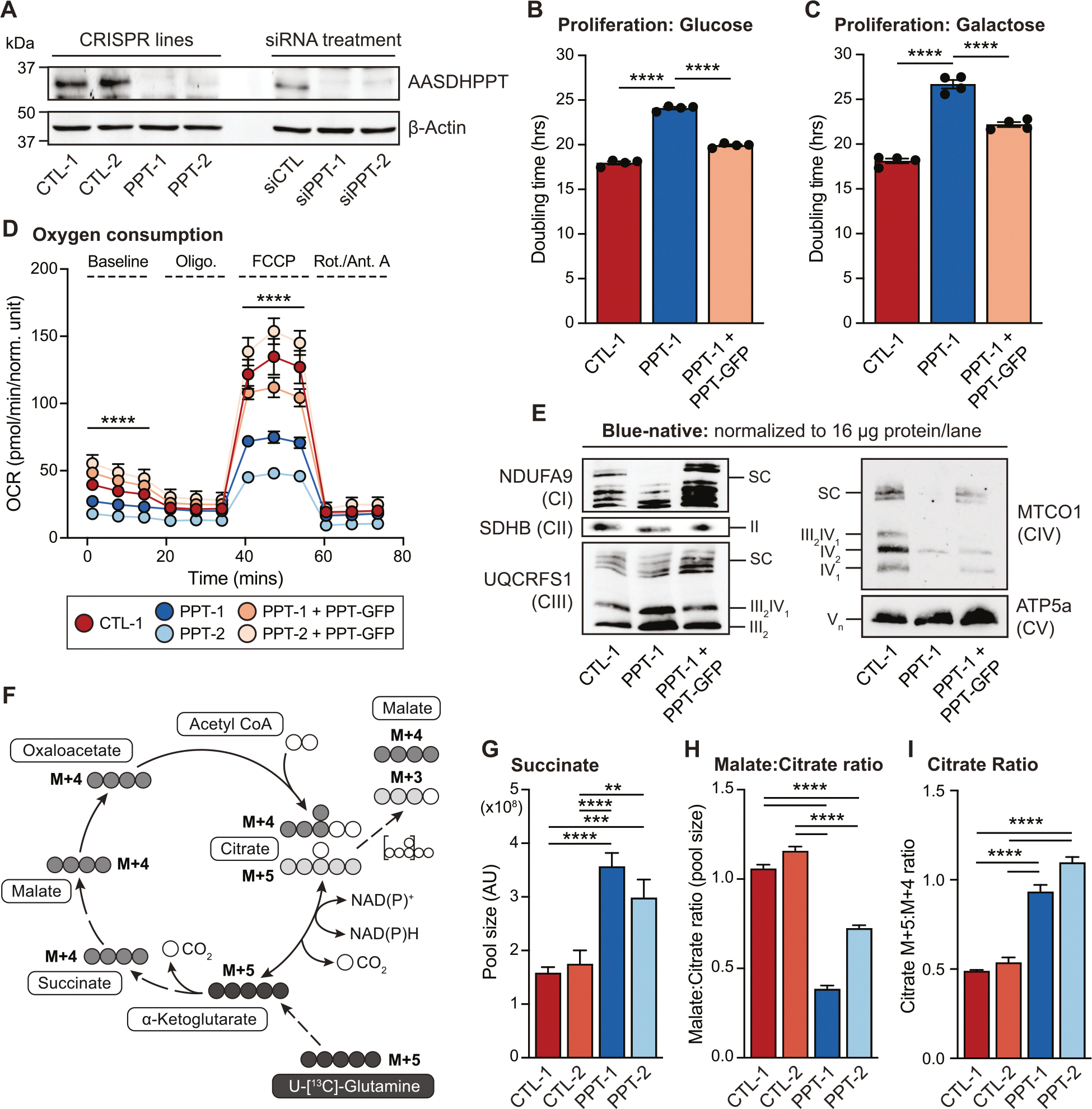
AASDHPPT is required for mitochondrial oxidative metabolism. (A) Whole cell lysates were separated by SDS-PAGE and immunoblotted for the denoted targets in clonal control, *Aasdhppt*-deficient, and siRNA-transfected cells. Data are representative of 3 biological replicates. (B–C) Cells were grown in either 25 mM glucose (B) or 10 mM galactose (C) in an IncuCyte^®^ system with images captured every 3 h for 4 days or 2 days respectively. Data represent the mean doubling time calculated from technical replicates of growth curves; error bars denote SEM **** = *p* < 0.0001. Data are representative of 1 experiment from *n* > 3 biological replicates. (D) Seahorse mitochondrial stress test for Oxygen Consumption Rate (OCR) of indicated cell lines expressing mtDSRed or PPT-GFP. Data are representative of 1 experiment from 3 biological replicates. Error bars represent SD. Significance determined at indicated time points using one-way ANOVA, **** = *p* < 0.0001 (E) Blue-native PAGE separation of isolated mitochondrial protein complexes from denoted cell lines expressing mtDSRed or PPT-GFP, followed by immunoblot with the indicated antibodies. Data are representative of n > 3 biological replicates. (F) Schematic of isotopomeric labeling patterns upon substitution of U^13^C-glutamine for unlabeled glutamine. Open circles indicate ^12^C carbons, where circles with grey, red, or blue, indicate ^13^C carbons. (G-H) Technical triplicate samples (*n* = 3) of the indicated cell lines cultured in U^13^C-glutamine for the indicated time points, harvested, and analyzed via LC-MS. Steady state metabolite pool sizes and ratios are shown for the 4-h labeling time point. (I) Ratio of Citrate M + 5 to Citrate M + 4 at the four-hour time point. Error bars represent SEM (B, C), SD (D, G, H, I). ** = *p* < 0.01, *** = *p* < 0.001, **** = p < 0.0001 as determined by ANOVA followed by Tukey’s multiple comparisons test.

To test whether PPT is required for mitochondrial oxidative metabolism, we used CRISPR/Cas-9 to mutate *Aasdhppt* in C2C12 mouse skeletal myoblasts. Complete loss of PPT was incompatible with growth; however, we generated two clonal cell lines with mutations in *Aasdhppt* (Supplementary Fig. 1A) that resulted in decreased PPT protein (PPT-1 and PPT-2, Fig. 1A), confirmed by siRNA-mediated knockdown (siPPT-1 and siPPT-2, Fig. 1A). Of note, C2C12 cells are near-tetraploid [5,6]. Consequently, we found three unique PPT alleles in each of our PPT-deficient cell lines (Supplementary Fig. 1A). In both cases, the top listed allele in Supplementary Fig. 1A was found about twice as frequently as the other two, likely indicating that there are two alleles with this sequence. Further, this analysis revealed that the PPT-2 cells still harbor a wild type allele, which corresponded to a low level of residual PPT protein expression, also visible by western blot (Fig. 1A and Supplementary Fig. 1A). PPT-2 thus often displays a milder phenotype when compared directly with PPT-1, even though both exhibit a loss of PPT protein (Fig. 1A).

We first asked whether PPT deficiency resulted in altered respiratory-dependent growth by culturing cells in media with either glucose or galactose, the latter requiring functional mitochondrial respiration. PPT-deficient cells showed significantly increased doubling times in both media conditions (Fig. 1B–C, Supplementary Fig. 1B–C). Next, we performed a Seahorse mitochondrial stress test and observed that PPT-deficient mutants exhibited reduced basal and maximal respiration (Fig. 1D, Supplementary Fig. 1D). To assess whether PPT-deficient respiration was associated with perturbed OXPHOS complexes, we performed blue-native PAGE and found that PPT-deficient mutants displayed reduced levels of electron transport chain (ETC) complex I (CI), complex II (CII) and complex IV (CIV). ATP synthase (CV) and complex III (CIII) were not affected by PPT-deficiency, but reductions in CIII-containing supercomplexes (SC) were observed (Fig. 1E, Supplementary Fig. 1E). All of these observations were rescued by re-expression of full length PPT with a C-terminal GFP-Flag tag (PPT-GFP) (Fig. 1B–E, Supplementary Fig. 1B–E).

ETC impairments are well known to affect mitochondrial metabolism and alter tricarboxylic acid (TCA) cycle flux [7–9]. We therefore reasoned that PPT-deficient cells may also display altered TCA metabolism. To assess TCA metabolism, we cultured cells in fully labeled U-[^13^C]-glutamine and evaluated TCA cycle intermediates for both steady state abundance and carbon contribution from labeled glutamine (Fig. 1F). PPT-deficient cells showed increased pool size of succinate and decreases in downstream metabolites, including fumarate, malate, and aspartate (Fig. 1G, Supplementary Fig. 1F–H). Citrate and α-ketoglutarate levels varied (Supplementary Fig. 1I and J); however, the ratio of malate to citrate was consistently reduced in PPT mutants (Fig. 1H). The reduction of TCA metabolites downstream of succinate relative to citrate suggests decreased oxidative TCA processing in PPT-deficient cells.

To assess whether this was indeed the case, we examined citrate labeling patterns from uniformly labeled glutamine. In general, M + 4 citrate results from the oxidation of glutamine, whereas M + 5 citrate results from the reductive carboxylation of glutamine (Fig. 1F). Increased reductive carboxylation is a common feature of respiratory-deficient cells [7,8]. In agreement with our pool size data, PPT-deficient cells showed reduced M + 4 citrate and increased M + 5 citrate compared to controls, indicating reduced oxidative TCA processing and increased reductive carboxylation of glutamine (Supplementary Fig. 1K). This is further supported by the increase in the ratio of citrate M + 5:citrate M + 4 at isotopic steady state (Fig. 1I). Accordingly, we also observed a decrease in downstream M + 4 malate (Supplementary Fig. 1L), likely due to a reduction in M + 4 citrate. In sum, the glutamine labeling patterns in PPT-deficient cells demonstrate increased reductive carboxylation of glutamine, which is typical of cells with ETC impairments^57,8^. Collectively, our data show that PPT mutant cells exhibit loss of oxidative mitochondrial metabolism.

### 2.2. AASDHPPT is required for mitochondrial fatty acid synthesis (mtFAS)

We next asked which mitochondrial target(s) of PPT were most prominently responsible for the observed mitochondrial phenotypes. As mentioned above, there are three known 4’PP-modified proteins in mammalian mitochondria, NDUFAB1, ALDH1L2, and DHRS2 [1]. To explore which of these putative targets might be responsible, we analyzed gene co-dependency relationships from the DepMap Public 23Q4+ Score Chronos dataset [10] to identify gene functionality within the same biological pathway [11]. Among the three known mitochondrial 4’PP-modified proteins, *AASDHPPT* (PPT) was positively correlated only with *NDUFAB1* (Pearson *r* = 0.24) (Supplementary Fig. 2A).

Unique among 4’PP-modified proteins, NDUFAB1 is a subunit of complex I of the ETC, and an integral part of the mitochondrial fatty acid synthesis (mtFAS) pathway. mtFAS builds de novo fatty acyl chains on NDUFAB1, which are required both as precursors for the biosynthesis of lipoic acid (LA), an important TCA cycle enzyme co-factor, as well as to stabilize ETC assembly factors via protein-protein interactions with NDUFAB1 [9,12]. Because mtFAS builds fatty acids on the terminal sulfhydryl group of the 4’PP PTM, the addition of a 4’PP to NDUFAB1 is required for successful mtFAS [13]. Of note, the mtFAS pathway gene *OXSM* ranked third out of all genes in the Depmap database for co-dependency with *AASDHPPT* (Pearson *r* = 0.42, Supplementary Fig. 2B) [14]. Additional mtFAS pathway genes also ranked highly, including *MCAT* and *MECR* (Pearson *r* = 0.36 and 0.32 respectively) (Supplementary Fig. 2C and D).

**Fig. 2.**
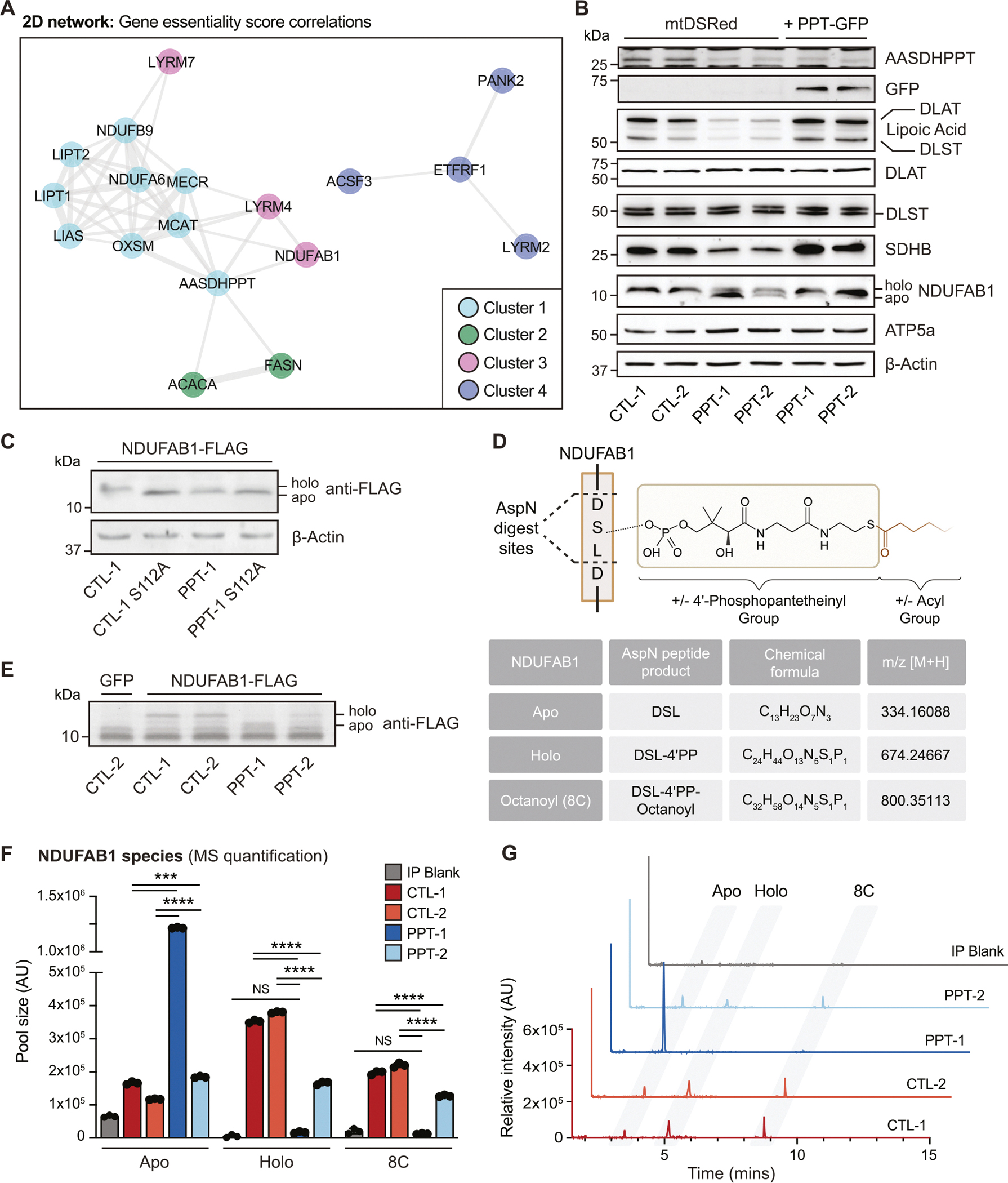
AASDHPPT is required for mitochondrial fatty acid synthesis pathway function. (A) Two-dimensional network diagram representing gene essentiality score correlations between selected genes associated with *AASDHPPT* and known substrate targets, mtFAS pathway members, LYRM family members, and the GO term “pantothenate metabolism” using a minimum threshold correlation score > 0.2. Different colors represent genes associated with clusters as shown in Supplementary Fig. 2E. Correlation strength is represented by the length and thickness of the connecting edge. (B) Whole cell lysates from indicated cell lines expressing mtDSRed or PPT-GFP fusion protein were separated by SDS-PAGE and immunoblotted for the denoted targets. Data are representative of 3 biological replicates. Red asterisk denotes ATP5a band from previous blot. (C) Whole cell lysates from indicated cell lines expressing either NDUFAB1-Flag or NDUFAB1_S112A_-Flag were separated by SDS-PAGE and immunoblotted for protein expression of the denoted targets. Data are representative of 3 biological replicates. (D) Schematic of mass spectrometry approach using AspN digestion to detect Apo-, Holo-, and Acyl-species of NDUFAB1. (E) Coomassie stain of SDS-PAGE gel of NDUFAB1-FLAG immunoprecipitation from the indicated cell line. (F) Relative quantification of NDUFAB1 AspN digest products via tandem liquid chromatography mass spectrometry *n* = 3 technical replicates from immunoprecipitation, representative of 3 distinct biological replicates. NS = not significant, *** = *p* < 0.001, **** = *p* < 0.0001 as determined by two-way ANOVA followed by Šidák’s multiple comparisons test. (G) Representative overlayed extracted ion chromatograms of Apo-, Holo-, and Acyl-NDUFAB1 from cells of the indicated genotype, quantified in F.

To further explore this relationship, we performed clustering of pairwise gene essentiality score correlations with *AASDHPPT*. We included genes associated with the GO term “pantothenate metabolism,” genes encoding known phosphopantetheinylated proteins, mtFAS pathway genes, and genes involved in downstream lipoic acid synthesis and ETC assembly, such as the leucine, tyrosine, arginine motif (LYRM) assembly factors which are regulated by mtFAS [9]. The analyzed genes grouped into four distinct clusters (Supplementary Fig. 2E). Two-dimensional mapping showed that *AASDHPPT* most strongly correlated with mtFAS pathway genes, as well as several LYRM family members (Fig. 2A). Taken together, these data suggested that impaired mitochondrial oxidative metabolism in PPT-deficient cells may be a downstream result of diminished mtFAS function, in agreement with the known roles of mtFAS [9,15].

To test this hypothesis, we examined whether PPT-deficiency altered known mtFAS pathway endpoints, including protein lipoylation and succinate dehydrogenase B (SDHB) stability, which is a reliable surrogate of the LYRM-dependent ETC assembly mechanism we previously described [9]. PPT-deficient cells exhibited reduced lipoylation of pyruvate dehydrogenase (PDH) and α-ketoglutarate dehydrogenase (OGDH) subunits (DLAT and DLST) along with reduced stability of SDHB (Fig. 2B), resembling the molecular phenotype of mtFAS-deficient cells. These findings also agree with our metabolic tracing data, which suggest reduced TCA cycle activity downstream of SDH (Fig. 1H, Supplementary Fig. 1F–H). PDH and OGDH lipoylation and SDHB stability were all rescued by transient re-expression of PPT-GFP compared to a control vector expressing mitochondrial DSRed (mtDSRed) (Fig. 2B).

Although one prior study demonstrated that PPT can add a 4’PP group to NDUFAB1 in vitro [2], its requirement for NDUFAB1 phosphopantetheinylation had never been tested in mammalian cells. We noted that NDUFAB1 migrated faster on SDS-PAGE gels in PPT-deficient cell lysates (Fig. 2B, Supplementary Fig. 2G), and hypothesized that this difference in migration might correspond to loss of the 4’PP, as unmodified apo-NDUFAB1 has previously been shown to migrate faster than 4’PP-modified holo-NDUFAB1 [2]. We tested this by mutating the modified serine to an alanine residue (S112A), thereby blocking phosphopantetheinylation of NDUFAB1. We expressed wild type NDUFAB1-Flag or NDUFAB1_S112A_-Flag in both control and PPT-deficient cells and found that NDUFAB1_S112A_-Flag in control cells migrates at the same molecular weight as NDUFAB1-Flag in PPT-deficient cells (Fig. 2C). Moreover, NDUFAB1_S112A_-Flag does not display any further downward shift in PPT-deficient cells, indicating that the two perturbations are not additive, and are both likely to be due to loss of 4’PP at S112 (Fig. 2C).

To directly assess whether phosphopantetheinylation of NDUFAB1 is decreased in PPT-deficient cells, we developed a method to detect 4’PP-modification of NDUFAB1 by liquid chromatography mass spectrometry (LCMS), borrowing from an assay developed in plant mitochondria and recently demonstrated for the first time in mammalian cell lines (Fig. 2D) [16,17]. We immunoprecipitated NDUFAB1-Flag from control and PPT-deficient cells and found that our isolated NDUFAB1 exhibited a similar gel shift as we had observed by blotting (Fig. 2E). We then digested these samples with an aspartate endoproteinase (AspN), resulting in a tripeptide (Asp-Ser-Leu) that we could monitor for 4’PP- or 4’PP-acyl modifications via LCMS. This allowed us to detect and quantify levels of apo-, holo-, and octanoyl-NDUFAB1 from control and PPT-deficient cells, verified by standards synthesized in vitro (Supplementary Fig. 2H). We found that relative to control cells, PPT-deficient cells have reduced levels of both holo- and octanoyl-NDUFAB1 (Fig. 2F and G). The PPT-1 clone displayed a striking accumulation of apo-NDUFAB1, in agreement with its stronger loss of function phenotype. In addition to the loss of holo-NDUFAB1, we found a modest increase in the levels of intracellular CoA in the PPT-1 and PPT-2 cell lines (Supplementary Fig. 2F). From these data, we conclude that PPT is required for 4’PP modification of NDUFAB1 and downstream mtFAS activity [15].

### 2.3. A pool of AASDHPPT localizes to the mitochondrial matrix

**Fig. 3.**
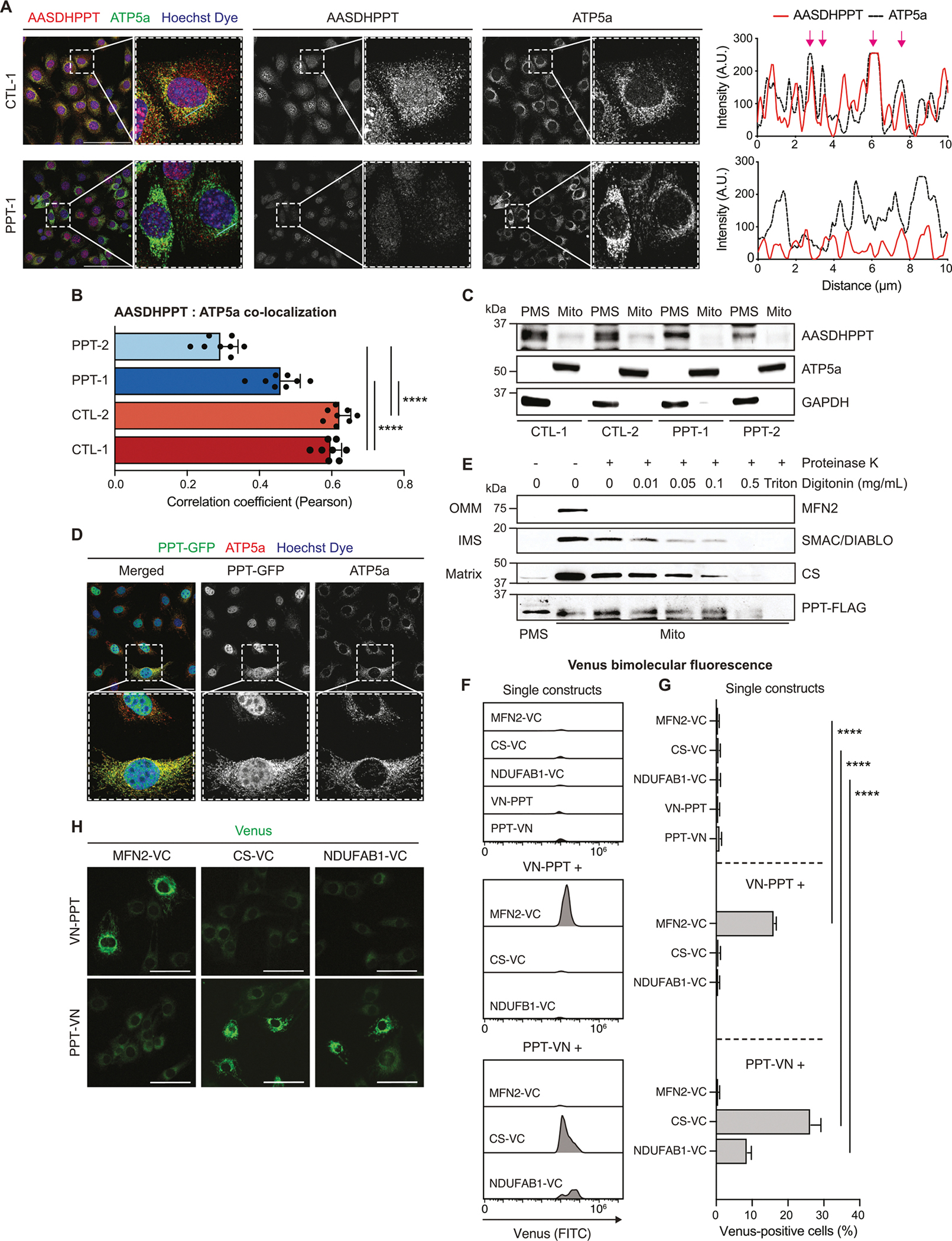
AASDHPPT localizes to the mitochondrial matrix. (A) Representative 40× HPF images of the indicated cell lines immunostained with Hoechst dye to visualize nuclei and antibodies against AASDHPPT (red) and ATP5a (green). Scale bars = 100 μm. Cyan arrows in merged images denote regions measured for fluorescent intensity line plots. Magenta arrows denote regions of prominent AASDHPPT and ATP5a signal overlap. (B) Quantification of AASDHPPT and ATP5a signal colocalization outside of the nucleus from *n* = 8 40× HPF per group assessed by Pearson’s correlation coefficient. (C) Subcellular fractionation of *Aasdhppt* mutant cell lines and control. Post-mitochondrial supernatant (PMS) and mitochondrial lysate (Mito) was isolated from the indicated cell lines and immunoblotted for denoted targets. Data are representative of 3 biological replicates. (D) Representative 40× HPF images of WT skeletal muscle myoblasts expressing AASDHPPT-GFP (PPT-GFP) stained with Hoechst dye and antibody against ATP5a (red). Scale bars represent 100 μm. Dashed outlines denote magnified regions shown in panels with solid outlines. For all statistical analysis data represent the mean; error bars denote SD. **** = *p* < 0.0001 as determined by one-way ANOVA with Šidák’s multiple comparisons test. (E) Proteinase K assay for submitochondrial localization. Mitochondria from cells expressing PPT-FLAG were incubated with increasing concentrations of digitonin and exposed to Proteinase K. PMS and mitochondria extracts were immunoblotted for the denoted targets. Data are representative of 3 biological replicates. (F) Representative histograms of Venus (FITC) fluorescence in the indicated BiFC cell lines. (G) Percentage of Venus-positive cells in the indicated cell lines as measured by flow cytometry. Data represent the mean; error bars denote standard deviation (n = 3 per group). Statistical significance was determined by one-way ANOVA with Tukey’s multiple comparison test. (H) Representative 20× images of endogenous Venus fluorescence in VC + VN BiFC cell lines. Scale bars represent 50 μm.

The question then became: if PPT is required for modification of mitochondrial proteins, does it act in the cytoplasm, prior to mitochondrial import, or is there an undescribed mitochondrial pool of PPT? To address this question, we performed immunofluorescence staining for endogenous PPT and mitochondrial ATP synthase subunit 5a (ATP5a) and assessed co-localization (Fig. 3A and B, Supplementary Fig. 3A–C). PPT staining exhibited a punctate, perinuclear expression pattern that co-localized with ATP5a in control cells (Pearson *r* = 0.60 and 0.62) (Fig. 3B) and was significantly reduced in PPT-deficient cells (Supplementary Fig. 3B). Additionally, PPT signal was surprisingly observed in the nuclei of some cells (Fig. 3A, Supplementary Fig. 3A).

To test whether this staining pattern was specific, we employed siRNA and found a similar reduction in endogenous PPT signal as before, although nuclear PPT signal was maintained despite knockdown (Supplementary Fig. 3D). We therefore also expressed PPT-Flag, stained for the Flag epitope, and found a similar punctate, peri-nuclear staining pattern that overlapped with citrate synthase but displayed no clear nuclear signal (Supplementary Fig. 3E). These results indicate that a sub-population of PPT localizes specifically to mitochondria but not to the nucleus. We further verified the observed mitochondrial localization using subcellular fractionation and Western blotting in control and PPT-deficient cells. We detected endogenous PPT expression in both mitochondria and post-mitochondrial supernatant (PMS) (Fig. 3C). PPT expression was reduced in both fractions, and nearly absent from mitochondria in PPT-deficient cells (Fig. 3C). We also characterized subcellular localization of PPT-GFP using immunofluorescence and found strong signal overlap with ATP5a (Fig. 3D), in agreement with our prior observations.

We then asked whether this pool of mitochondrially-localized PPT was mitochondrially associated or in the mitochondrial matrix with NDUFAB1 and the rest of the mtFAS machinery. To tackle this question, we performed a proteinase K (PK) protection assay. In this assay, mitochondrial proteins are differentially vulnerable to PK digestion based on their sub-mitochondrial localization. Outer mitochondrial membrane (OMM) proteins are always accessible to PK, and upon treating the mitochondria with increasing concentrations of digitonin, intermembrane space (IMS) become exposed, while matrix proteins remain the most protected until addition of triton. Using Mitofusin 2 (MFN2), SMAC/DIABLO, and citrate synthase (CS) as markers of the OMM, IMS, and matrix, respectively, we performed the PK protection assay and blotted for endogenous PPT. In agreement with our fractionation data in Fig. 3C, we observed a faint mitochondrial PPT band, suggesting matrix localization of mitochondrial PPT (Supplementary Fig. 3F). Because endogenous PPT shows relatively weak mitochondrial signal via blotting, we performed the same assay with a C-terminal Flag tagged PPT construct, with similar results (Fig. 3E). To further verify these findings, we employed a bimolecular fluorescence complementation (BiFC) system [18]. We used a split-Venus tag and attached the N-terminus of the Venus fluorophore (VN, residues 1–172) to either the N- or C-terminus of PPT, while the C-terminus of Venus (VC, residues 155–238) was attached to the C-terminus of a control protein with known localization. This system produces fluorescent signal only when both halves of the split-Venus tag are able to associate within a compartment [18]. For control proteins, we again chose MFN2 and CS, along with NDUFAB1 itself. We observed significant Venus signal only when PPT-VN cells were co-expressed with either CS-VC or NDUFAB1-VC, indicating that PPT localizes to the mitochondrial matrix (Fig. 3F–H, see Supplementary Fig. 3G for gating strategy).

### 2.4. AASDHPPT routes to mitochondria via an N-terminal targeting sequence

Most mitochondrial proteins are encoded by nuclear genes and imported into mitochondria via an N-terminal mitochondrial targeting sequence (MTS), consisting of approximately 20 to 40 amino acid residues [19]. We noticed that in cells expressing N-terminally tagged PPT (VN-PPT), positive Venus fluorescence was detected when co-expressed with MFN2-VC (Fig. 3E–G), indicating that N-terminally tagged VN-PPT is mitochondrially associated on the OMM. Given this finding, we hypothesized that mitochondrial localization of PPT may be driven by an N-terminal MTS.

**Fig. 4.**
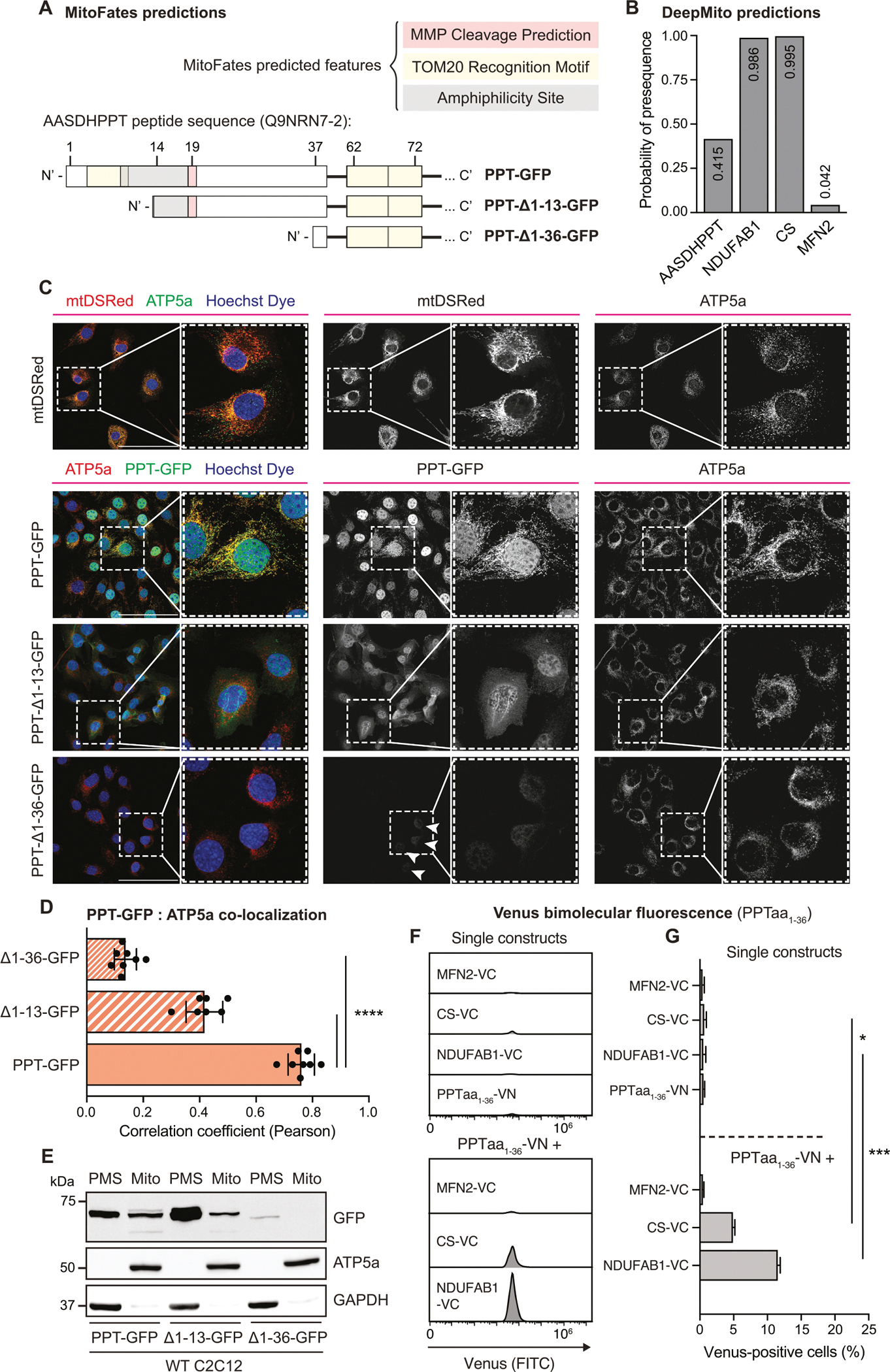
AASDHPPT has a N-terminal MTS. (A) Schematic Illustration of MitoFates [20] predicted features in full length AASDHPPT (PPT-GFP) and AASDHPPT with a 1–13 (Δ1–13) or 1–36 (Δ1–36) N-terminal truncation. (B) Quantification of MTS presequence probability in AASDHPPT, NDUFAB1, Citrate Synthase (CS) and Mitofusin 2 (MFN2) determined by MitoFates. (C) Representative 40× HPF images of WT cells expressing mtDSRed, PPT-GFP, 13 residue N-terminal truncation of PPT (PPT_Δ1–13_-GFP), or 36 residue N-terminal truncation of PPT (PPT_Δ1–36_-GFP) constructs, stained with Hoechst dye and antibody against ATP5a (red). Scale bars represent 100 μm. Arrows denote weak expression of GFP signal detected. (D) Quantification of PPT-GFP and ATP5a signal colocalization outside of the nucleus from n = 8 40× HPF assessed by Pearson’s correlation coefficient from experiment shown in (C). Data represent the mean; error bars denote SD. **** = p < 0.0001, determined by one-way ANOVA with Dunnett’s multiple comparisons test. (E) Subcellular fractionation of WT cells expressing PPT-GFP, PPT_Δ1–13_-GFP, or PPT_Δ1–36_-GFP. Post-mitochondrial supernatant (PMS) and mitochondrial lysate (Mito) was isolated from the denoted cell lines, separated by SDS-PAGE, and immunoblotted for the indicated targets. Data are representative of 3 biological replicates. (F) Representative histograms of Venus (FITC) fluorescence in the indicated BiFC cell lines. (G) Percentage of Venus-positive cells in the indicated cell lines as measured by flow cytometry. Data represent the mean; error bars denote standard deviation (n = 3 per group). Statistical significance was determined by one-way ANOVA with Tukey’s multiple comparison test. **** = *p* < 0.0001.

To explore this hypothesis, we analyzed AASDHPPT using the MitoFates [20] and DeepMito [21,22] prediction algorithms. MitoFates reported a 41.5 % probability of a mitochondrial presequence at the N-terminus of PPT, with a TOM20 recognition motif from amino acids 5–9 and an MPP cleavage site at amino acid 19 (W) (Fig. 4A–B). Alternatively, DeepMito predicted a 47 % probability of mitochondrial localization. This region of the amino acid sequence is well conserved among mammals (Supplementary Fig. 4A). To test whether the N-terminus of PPT is a bonafide MTS, we generated N-terminal truncations to the next two methionines at positions 14 and 37 (PPT_Δ1–13_-GFP and PPT_Δ1–36_-GFP, respectively, Fig. 4A) and examined their localization using fluorescence microscopy. While PPT-GFP co-localized strongly with ATP5a, PPT_Δ1–13_-GFP exhibited diffuse localization throughout the cell (Fig. 4C–D). PPT_Δ1–36_-GFP exhibited weak fluorescent signal mostly within the nucleus, suggesting that this larger N-terminal truncation destabilizes PPT (Fig. 4C–D). To further refine the MTS based on the MitoFates prediction, we also tested an N-terminal truncation replacing the first 20 amino acids with a single methionine (PPT_Δ1–20_-GFP). PPT_Δ1–20_-GFP behaved similarly to PPT_Δ1–36_-GFP, with very low levels of diffuse expression (Supplementary Fig. 4B). Sub-cellular fractionation and Western blotting supported our microscopy results: while PPT-GFP and PPT_Δ1–13_-GFP are both present in PMS and mitochondrial fractions, PPT_Δ1–13_-GFP accumulates in the PMS (Fig. 4E), suggesting difficulty in localizing to mitochondria.

We also performed the converse experiment, in which we added the first 36 amino acids of PPT to GFP, to test the sufficiency of the putative MTS to drive GFP to the mitochondria (PPT MTS-GFP). PPT MTS-GFP showed strong overlap with ATP5a staining as well as strong expression in the mitochondrial fraction via Western blot (Supplementary Fig. 4C and D). We took a parallel BiFC approach using the split-Venus tag system, expressing the first 36 amino acids of PPT fused to the N-terminus of Venus (PPTaa_1–36_-VN) with either MFN2-VC, CS-VC, or NDUFAB1-VC. As with PPT-VN, significant Venus signal was only observed when PPTaa_1–36_-VN was co-expressed with either CS-VC or NDUFAB1-VC (Fig. 4F and G, Supplementary Fig. 4F), indicating that the first 36 amino acids of PPT are sufficient to target proteins to the mitochondrial matrix.

### 2.5. Mitochondrial localization is required for function of AASDHPPT in oxidative metabolism

**Fig. 5.**
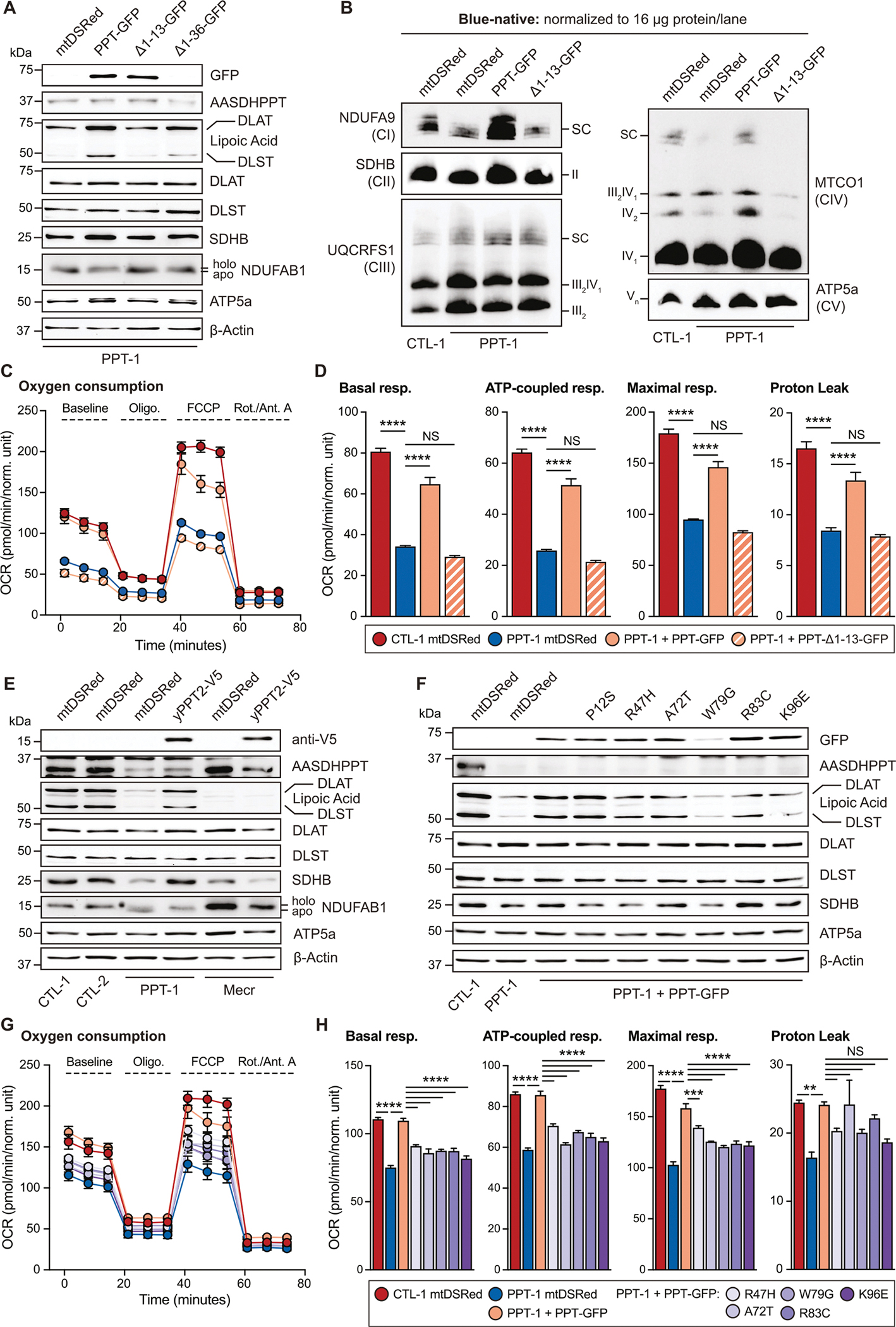
Mitochondrial localization of AASDHPPT is required for mtFAS function. (A) Whole cell lysates from cells expressing mtDSRed, PPT-GFP, PPT_Δ1–13_-GFP, or PPT_Δ1–36_-GFP were separated by SDS-PAGE and immunoblotted for the denoted targets. Data are representative of *n* > 3 biological replicates. (B) Blue-native PAGE separation of isolated mitochondrial protein complexes from denoted cell lines expressing mtDSRed, PPT-GFP, or PPT_Δ1–13_-GFP, followed by immunoblot with the indicated antibodies. Data are representative of n > 3 biological replicates. (C) Seahorse mitochondrial stress test for Oxygen Consumption Rate (OCR) of indicated cell lines expressing mtDSRed, PPT-GFP, or PPT_Δ1–13_-GFP. Data are representative of 1 experiment from 3 biological replicates. Error bars represent SD. (D) Quantification of basal respiration, ATP-production coupled respiration, maximal respiration and proton leak from data in (C). Data are mean OCR; error bars are SEM. **** = p < 0.0001, determined by one-way ANOVA with Tukey’s multiple comparisons test. (E) Whole cell lysates from clonal control, *Aasdhppt*-, and *Mecr*-deficient cells expressing mtDSRed or yPpt2-V5 were separated by SDS-PAGE and immunoblotted for the denoted targets. Data are representative of 3 biological replicates. (F) Whole cell lysates from the indicated cell lines expressing mtDSRed, PPT-GFP P12S, PPT-GFP R47H, PPT-GFP A72T, PPT-GFP W79G, PPT-GFP R83C, or PPT-GFP K96E were separated by SDS-PAGE and immunoblotted for the denoted targets. Immunoblots are representative of 3 biological replicates. (G) Seahorse mitochondrial stress test for Oxygen Consumption Rate (OCR) of indicated cell lines expressing mtDSRed, PPT-GFP, PPT-GFP R47H, PPT-GFP A72T, PPT-GFP W79G, PPT-GFP R83C, or PPT-GFP K96E. Data are representative of 1 experiment from 3 biological replicates. Error bars represent SD. (H) Quantification of basal respiration, ATP-production coupled respiration, maximal respiration and proton leak from data in (G). Data are mean OCR; error bars are SEM. ** = *p* < 0.01, *** = *p* < 0.001, **** = p < 0.0001, determined by one-way ANOVA with Šidák’s multiple comparisons test.

To test whether this newfound mitochondrial localization is required for PPT to modify mitochondrial targets, we transduced PPT-deficient cells with mtDSRed, PPT-GFP, PPT_Δ1–13_-GFP, or PPT_Δ1–36_-GFP, and assessed mtFAS pathway activity. Expression of PPT-GFP rescued protein lipoylation and SDHB stability, whereas PPT_Δ1–13_-GFP and PPT_Δ1–36_-GFP both failed to rescue either of these mtFAS-dependent endpoints, nor did they rescue NDUFAB1 migration (Fig. 5A, Supplementary Fig. 5A). We also performed BN-PAGE and Seahorse mitochondrial stress tests as before with control and PPT-deficient cells expressing mtDSRed, PPT-GFP, or PPT_Δ1–13_-GFP. We found that PPT_Δ1–13_-GFP rescued ETC complex levels, along with basal and maximal oxygen consumption rates, less well than the full length PPT-GFP construct (Fig. 5B–D, Supplementary Fig. 5B–D).

Next, we took advantage of the *Saccharomyces cerevisiae* mitochondrial PPTase, yPpt2, (which specifically resides in the matrix) to test if 4’PP mitochondrial modifications rescue the mtFAS-associated mitochondrial defects observed in PPT mutant cells. Indeed, yPpt2-V5 localizes strongly to mitochondria (Supplementary Fig. 5E) and efficiently restores protein lipoylation, SDHB levels, and NDUFAB1 migration in PPT-deficient cells (Fig. 5E). Notably, yPpt2-V5 expression does not rescue protein lipoylation or SDHB in cells deficient for the mtFAS enzyme MECR, indicating that this rescue cannot compensate for impairments to mtFAS downstream of PPT (Fig. 5E, Supplementary Fig. 5F). Together with our truncation mutant analyses, these data demonstrate that mitochondrial localization of PPT is necessary and sufficient for phosphopantetheinylation of NDUFAB1, mtFAS pathway activity, ETC assembly and mitochondrial respiration.

### 2.6. Identification of patient variants of AASDHPPT with reduced function

We recently described a pathogenic variant in the mtFAS gene *MCAT* c.812 T > C; p.T271I (rs760294168; NM_173467.4) [23]. As our data demonstrate that perturbed *Aasdhppt* expression results in similar phenotypes as mtFAS loss, we wondered whether there may be patients with pathological variants in *AASDHPPT*. We searched the ClinVar database [24] and identified several missense variants of uncertain significance (VUS) in *AASDHPPT* that were associated with suspected genetic disease. We selected six of these variants for analysis: c.343C > T; p.P12S (NM_015423.3), c.140G > A; pR47H (NM_015423.3), c.214G > T; pA72T (NM_015423.3), c.235 T > G; pW79G (NM_015423.3), c.247C > T; pR83C (NM_015423.3), and c.286 A > G; pK96E (NM_015423.3).

We created each of these variants and assessed their ability to rescue mtFAS endpoints in our PPT-deficient cells. We found that all variants, except for PPT-GFP P12S, exhibited weaker rescue than wild type PPT (Fig. 5F–H, Supplementary Fig. 5G). Of note, cells expressing the PPT-GFP W79G variant showed low levels of protein expression compared to the other variants (Fig. 5F, Supplementary Fig. 5G–H) despite expression from the same promoter, suggesting that this variant may result in protein instability. These data identify R47H, A72T, W79G, R83C, and K96E as potential clinically observed pathologic variants of *AASDHPPT*, with molecular phenotypes attributable to altered mtFAS activity and perturbed cellular respiration.

## 3. Discussion

Our data are the first to position PPT as a critical mediator of mammalian mitochondrial oxidative metabolism, due to its requirement for proper function of the mtFAS pathway. Although prior work showed that PPT was capable of phosphopantetheinylating NDUFAB1 in vitro [2], its requirement had never been tested in cells. While we show definitively that mitochondrial PPT is required for phosphopantetheinylation of NDUFAB1 and downstream activity of the mtFAS pathway, ETC function, and oxidative metabolism, we did not explore its involvement in 4’PP-modification of ALDH1L2 and DHRS2 in the present study. Future work focused on the role of PPT in these additional pathways will be important to expand our understanding of the full scope of PPT action in cells.

Our data also demonstrate that PPT is targeted to mitochondria via an N-terminal MTS. However, while our data strongly support matrix localization of AASDHPPT, we cannot fully exclude the possibility that a fraction of the protein associates with the outer mitochondrial membrane, where it may also play important role(s). Despite this possibility, because the Δ1–13 mutant fails to rescue respiration or lipoylation, we predict that any OMM-associated pool is unlikely to be functional in the mtFAS pathway.

Other intriguing questions about the localization of PPT remain unanswered. For example, how the mitochondrial localization of PPT is controlled in cells to produce two distinct pools is completely unstudied. The discovery of distinct pools of PPT also raises questions about what factors control PPT activity in each compartment. For instance, PPT uses CoA as the substrate for 4’PP-modification of target proteins. How perturbations in CoA availability affect PPT enzymatic activity may have important implications for PPT targets such as mtFAS in diseases with CoA dysregulation [25]. Future work aimed at uncovering whether PPT localization is dynamic and how PPT activity is differentially regulated within the mitochondria and cytoplasm will also be interesting and important areas for study.

Of note, we identified five probable pathogenic patient variants in *AASDHPPT*. Our results predict that patients harboring these mutant alleles likely exhibit impaired mtFAS, leading to diminished mitochondrial function. These data add to the growing list of patients with pathogenic variants in mtFAS and lipoic acid synthesis genes and support the notion that impairments in CoA metabolism may cause disease via mtFAS dysfunction [26,27]. Taken together, this work connects PPT activity within mitochondria to oxidative metabolism, and identifies a new group of potential related pathologies.

## 4. Methods

### 4.1. Cell culture

C2C12 immortalized mouse skeletal myoblasts (ATCC CRL-1772, verification provided by ATCC) were grown in DMEM with 4.5 g/L glucose, 4 mM glutamine, and sodium pyruvate (Corning, 10–013-CV) and 10 % FBS (Sigma, F0926) at 37 °C, 5 % CO_2_.

#### 4.1.1. Generation of Aasdhppt mutant cell lines

Four sgRNA sequences (Table 1) were designed targeting exon 1 or exon 2 of the mouse *Aasdhppt* gene and subcloned into the pLentiCRISPRv2 [28]. Parental C2C12s were transfected with pLentiCRISPRv2_sgAasdhppt or empty vector using JetOptimus reagent (Polyplus, 76299–630). 48 h after transfection, single GFP+ cells were sorted into 96-well plates to obtain clonal lines. Clones were screened for AASDHPPT protein expression; two *Aasdhppt* mutant clones and two controls were selected for further experiments. While PPT-deficient clones were derived from both guide RNAs, clones with the strongest loss of PPT expression were derived from sgRNA 1, and we focused on these clones.

#### 4.1.2. siRNA knockdown

To knockdown *Aasdhppt* or *Ndufab1*, siRNAs were acquired as indicated in (Table 2). siRNAs were packaged with Lipofectamine RNAiMAX, transfected into C2C12, and cells were cultured for 72 h post-transfection before harvest. ON-TARGETplus Non-targeting Control Pool (D-001810–10-05) was used as a negative control.

#### 4.1.3. Retroviral expression

For localization and rescue experiments, control and *Aasdhppt* mutant lines were transiently infected with control retrovirus (pQXCIP mtDSRed) or pMMLV retrovirus vectors purchased from VectorBuilder (Chicago, IL, USA), harboring full length or N-terminal truncations of AASDHPPT with C-terminal GFP and Flag tags, or yPPT2 with C-terminal Flag tag. *Site-directed mutagenesis.* For generation of AASDHPPT-GFP-Flag point mutations, primers in Table 3 were used with a New England Biolabs Q5 Site-Directed Mutagenesis Kit.

#### 4.1.4. Growth assays

For growth assays 5000 cells/well were plated in 24-well plates and grown in DMEM with 10 % FBS, 4 mM glutamine, and 25 mM glucose for 4 days or 20 % FBS, 4 mM glutamine, and 10 mM galactose for 2 days in technical quadruplicates. Percent confluency was measured in an IncuCyte^®^ chamber (Sartorius) from sixteen images at 10× magnification every 3 h. Percent confluency curves were fit with a non-linear regression during the logarithmic growth phase using the exponential growth equation in GraphPad Prism to determine population doubling time.

### 4.2. Genomic sequence analysis of AASDHPPT mutants

Genomic DNA was extracted from each clonal mutant cell line using a column-based kit (Qiagen 69,504). *Aasdhppt* was amplified using primers described in Table 4, TOPO-cloned (Thermo Fischer), and sanger sequenced. Sequences were analyzed using Snapgene software.

### 4.3. Crude mitochondrial isolation

Cells were harvested and processed as previously described [9]. Briefly, cell pellets were resuspended in CP-1 buffer (100 mM KCl, 50 mM Tris-HCL, 2 mM EGTA, pH 7.4) with mammalian protease inhibitor cocktail (mPIC, Millipore Sigma P8340), mechanically lysed, and centrifuged to pellet unlysed cells and debris. Supernatant was transferred to a new tube and centrifuged at 10,000 ×*g* for 10 min to pellet crude mitochondria. Post-mitochondrial supernatant (PMS) was used for Western blot or discarded. Mitochondrial pellets were resuspended in a small volume of either RIPA buffer (10 mM Tris-HCl, pH 8.0, 1 mM EDTA, 0.5 mM EGTA, 1 % Triton X-100, 0.1 % Sodium Deoxycholate, 0.1 % SDS, 140 mM NaCl) with mPIC, or CP-1 buffer. Resuspended pellets were used for applications described below.

### 4.4. SDS-PAGE and immunoblotting

Whole cell lysate, PMS, and/or crude mitochondrial fractions were normalized for total protein content via Pierce BCA Protein assay (Thermo Fisher Scientifc 23,225). Samples were resolved by SDS-PAGE and transferred to nitrocellulose. Immunoblotting was performed using the indicated primary antibodies (Table 5) with visualization of protein signal using Rabbit and Mouse IgG Antibody DyLight 680 or 800 secondary antibodies and Bio-Rad ChemiDoc Imaging System. Alternatively, secondary anti-mouse or anti-rabbit HRP antibody and SuperSignal West Femto Maximum Sensitivity Substrate (Thermo Fisher Scientific 34,096) were used to visualize band signals.

Mitochondrial sublocalization was performed by treating 20 μg of isolated crude mitochondria with 20 μg/mL Proteinase K (NEB P8107S) in the presence of ascending concentrations of digitonin (0–0.5 mg/mL) for 15 min on ice. Protease digestion was stopped by the addition of mPIC. PMS and mitochondria extracts were then subjected to SDS-PAGE as above.

### 4.5. Seahorse assay

Oxygen consumption assays were performed on a Seahorse XFe96 Analyzer (Agilent). Assays were performed in seahorse media (Agilent 103,680) with 25 mM glucose, 2 mM glutamine, and 1 mM sodium pyruvate. Standard mitochondrial stress tests were conducted using 1 μM oligomycin, 3 μM FCCP, and 0.5 μM Rotenone +0.5 μM Antimycin A. Measurements were taken over 3 min, with three measurements per phase. Data were normalized to crystal violet staining for approximation of cell number or by measurement of well % confluency using an IncuCyte^®^ chamber. Results were analyzed in WAVE software and the Agilent Seahorse Analytics browser-based application.

### 4.6. Blue native-PAGE

Crude mitochondria pellets were resuspended in 1× pink lysis buffer (Invitrogen BN2003). Digitonin (GoldBio D-180–2.5) was added to samples at a final concentration of 1 % mass/volume and samples were incubated on ice for 15 min. Insoluble material was pelleted and supernatant was transferred to new tubes. NativePAGE sample buffer (Invitrogen BN2004) was added and samples were run on 3–12 %, Bis-Tris NativePAGE gels (Invitrogen BN1001BOX) with NativePAGE anode buffer (Invitrogen 2001) and dark blue cathode buffer (Invitrogen BN2002) according to manufacturer’s guidelines, then transferred to PVDF membranes. Following transfer, membranes were washed in 8 % acetic acid solution, followed by 100 % methanol and then sterile distilled water to remove residual Coomassie. Membranes were then blocked in 5 % BSA at room temperature and incubated with the indicated primary antibodies overnight at 4 °C (Table 5). Secondary anti-mouse or anti-rabbit HRP antibody and SuperSignal West Femto Maximum Sensitivity Substrate (Thermo Fisher Scientific 34096) was used to visualize bands with a Bio-Rad ChemiDoc Imaging System.

### 4.7. Glutamine tracing

Cell lines were plated in triplicate in 6-well plates and allowed to adhere for 2 h, at which point the media was changed to unlabeled tracing media (Corning DMEM 17–207-CV with 1 mM sodium pyruvate, 5 mM glucose, and 4 mM glutamine). Wells were changed to labeled media at the indicated time points prior to harvest (Corning DMEM 17–207-CV with 1 mM sodium pyruvate, 5 mM glucose, and 4 mM U^13^C-glutamine). Three wells per cell line were left unlabeled. At time of harvest, plates were washed with normal saline and frozen at −80 °C. Plates were extracted for metabolites using 40:20:20 methanol:acetonitrile:water scaled to the number of cells. Lysates were sonicated and incubated on ice for 60 min, then insoluble material was pelleted. 1000 μL of supernatant was dried using a SpeedVac concentrator.

### 4.8. Liquid chromatography – mass spectrometry (LC-MS)

Dried metabolomics extracts were resuspended in 100 % water containing 25 μg/mL D5-Glutamate (DLM-556, Cambridge). 2 μL of resuspended samples were injected on column. Data were collected on a Vanquish liquid chromatography system coupled to an Orbitrap Exploris 240 (Thermo Fisher Scientific) using a heated electrospray ionization (H-ESI) source in ESI negative mode. All samples were run through a 24-min reversed-phase chromatography ZORBAX extend-C18 column (1.8 μm, 2.1 mm × 150 mm, 759700–902, Agilent, California, USA) combined with a guard column (1.8 μm, 2.1 mm × 5 mm, 821725–907, Agilent). Full scan data were collected with a scan range of 70–800 *m*/*z* at a mass resolution of 240,000. Fragmentation data was collected using a data-dependent MS2 (ddMS2) acquisition method with MS1 mass resolution at 120,000, MS2 mass resolution at 15,000, and HCD collision energy fixed at 30 %. Natural isotope abundance correction was done using the FluxFix Isotopologue Analysis Tool*14* (version 0.1) web-based application [29]. Data analysis was conducted in Skyline (version 23.1.0.268).

### 4.9. AASDHPPT co-essentiality analysis and network modeling

Gene essentiality scores and gene co-dependency Pearson correlation values were obtained from the DepMap Portal Project Achilles 23Q4 release [14,30–32]. Co-Dependency plots were generated from gene essentiality scores obtained directly from the Portal. For two-dimensional network analysis, gene essentiality correlations for indicated genes were used to create a data matrix. From this matrix, methodologies and code originally published by Arnold et al. [33] and available at: https://github.com/finley-lab/coessentiality-network were used to generate a correlation matrix heatmap of codependent gene modules. Co-dependent modules were then visualized as a network diagram and genes with low correlation scores (Pearson *r* < 0.2) filtered out as previously described [33]. Graph edges were weighted according to the strength of pairwise correlations.

### 4.10. Relative quantification of NDUFAB1 species

Method was adapted from [16,17]. Standards for NDUFAB1 modifications were synthesized as in^16^ and digested with AspN (Sigma, 11420488001) overnight at 37 °C in a ratio of 1:20 (*w*/w) enzyme to sample, buffered to a pH of 7.6 with MOPS, then quenched by addition of methanol. Digested standards were pooled for detection by LC-MS. Cells from each genotype were transiently transfected with either NDUFAB1-Flag or empty vector control. After 48 h cells were harvested and lysed with triton lysis buffer (1 % triton, 10 mM HEPES, 0.3 mM EDTA, 120 mM NaCl, and mPIC). Samples were then spun to pellet debris and cleared supernatants were incubated with anti-FLAG magnetic beads (Sigma, M8823). Bound proteins were eluted from beads using Flag peptide (Sigma, F4799) in elution buffer (10 mM HEPES, 50 mM NaCl, mPIC), filtered, and quantified via Bradford assay (BioRad, 5000205). Protein that remained bound to beads was eluted with 1× Laemmli buffer and run on an SDS-PAGE gel for Coomassie staining. 3 μg of eluted protein from each sample were digested with AspN as above. Samples were dried, resuspended in 50 % methanol:50 % water, and analyzed with a Vanquish (Thermo Scientific) liquid chromatography system coupled to an Orbitrap ID-X Tribrid mass spectrometer (Thermo Scientific) in full scan mode for standards and with targeted simulated ion monitoring (tSIM) for immunoprecipitation samples. A reversed-phase C18 column (Agilent ZORBAX RRHS Extend-C18 Column, 759700–902) was used to separate samples using acidic (0.1 % formic acid) solvents. The first 1.5 min of column eluants were diverted to waste due to the presence of MOPS buffer in this time frame. Chromatographic traces and peak areas were quantified from extracted ion chromatograms, matched to retention times from relevant standards using FreeStyle software (Thermo).

### 4.11. Immunostaining and microscopy

Cells were grown in chambered cover glass slides and fixed in cold 4 % paraformaldehyde (PFA) in PBS. Cells were permeabilized and blocked in PBS with 0.5 % BSA, 5 % goat serum, and 0.3 % Triton X-100, then incubated with the indicated primary antibodies followed by washes with PBS prior to incubation with Alexa Fluor conjugated secondary antibodies (Table 5). Cells were then stained with Hoechst Dye and imaged on a Zeiss LSM 880 with Airyscan and non-linear optics, using a 40× water objective and 1.2 NA to collect z-stacks, or with a Nikon Eclipse T*i*2 epifluorescent microscope (20× objective, 0.75 NA or 60× oil immersion objective, 1.40 NA).

For quantification of endogenous AASDHPPT expression, regions of interest (ROIs) were manually drawn with Fiji software then measured for mean fluorescent intensity (MFI) in the AASDHPPT channel. For statistical analysis, GraphPad Prism v10 software was used to remove outliers from the data set by the ROUT method, Q = 1 %. For colocalization analysis, Hoescht nuclear signal was first used to subtract nuclear regions so that colocalization analysis would be limited to extranuclear organelles and cytoplasmic regions. Merged channels were then processed by the BIOP JACoP Fiji plugin (https://c4science.ch/w/bioimaging_and_optics_platform_biop/image-processing/imagej_tools/jacop_b/) using the Otsu method of thresholding for each channel to generate Pearsons Correlation values.

### 4.12. Bimolecular fluorescence complementation (BiFC) assay

For BiFC experiments, five split-Venus constructs were designed and synthesized by VectorBuilder in a pMMLV retroviral backbone. Wildtype C2C12 cells were infected with PPT-VN or VN-PPT in combination with each VC construct or an empty vector control (PQXCIP for VC constructs, and PQXCIB for VN constructs). Venus fluorescence in each line was assessed on an Accuri bench-top cytometer and analyzed using FlowJo (v. 10.10.0). A representative gating strategy is shown in Supplementary Fig. 3C. Visual confirmation of fluorescence was achieved by imaging all BiFC cell lines on a Nikon Eclipse Ti2 microscope.

### 4.13. Statistics

Statistical analysis was performed using GraphPad Prism 10. Unless otherwise noted, data were analyzed by one- or two-way ANOVA as appropriate followed by two-sided Dunnett’s multiple comparison test (when compared to only control) or Šídká’s or Tukey’s multiple comparison test (when comparing all groups). A *p*-value of <0.05 was considered statistically significant.

## Supplementary Material

MMC5

MMC2

MMC3

MMC4

MMC1

Supplementary data to this article can be found online at https://doi.org/10.1016/j.metabol.2025.156413.

## Figures and Tables

**Table 1:** Aasdhppt guide RNA sequences.

sgRNA cloned into pLentiCRISPRv2	Sequence

PPT 1	GATGCGGTCTATCCAGCCGG
PPT 2	GCAGACGGATATGATCCCAA
PPT 3	AGCGGACCGAGCCGGTCGTA
PPT 4	GGAACACCATACGACCGGCT

**Table 2:** siRNA sequences.

siRNA target	ID#	Sequence

*Aasdhppt* #1	Thermo Fisher Scientific s85366	CGUUGAAUCCAUAUCCCAAtt
*Aasdhppt* #2	Thermo Fisher Scientific s85364	GGACUCAGCUGGAUAUGUUtt
*Ndufab1 Pooled*	Horizon Discovery L-055864-01-0005	CACUGACGUUAGACGGAAU
		CAGAAAAGCUCUCCGUAAA CUGAAGAAUUGGCGCAAAA GGAUGUGUAUGAAUAAAGU

**Table 3:** Primer sequences used for site directed mutagenesis generation of patient variant constructs.

Primers for site-directed mutagenesis	sequence

P12S Fwd	GTGCGTGGTGAGCTCCATGGAGG
P12S Rev	AGCCTCTTAGCGGGG
R47H Fwd	GGAGAAGGAGCATATCGGCAAGTTC
R47H Rev	TCCGGCTGGATAGAC
A72T Fwd	GAAATTAGTTACCGAGAAATTGAATATCCC
A72T Rev	CTTATCATCAAACGACCAG
W79G Fwd	GAATATCCCTGGCGATCATATCCGTC
W79G Rev	AATTTCTCTGCAACTAATTTC
R83C Fwd	GGATCATATCTGCCTGCAGAGAAC
R83C Rev	CAAGGGATATTCAATTTCTC
K96E Fwd	GGTTCTTGCGGAAGACTCGTTGA
K96E Rev	GGCTTTCCCTTTGAAGTTCTC
NDUFAB1 S112A Fwd	GGGCTTAGACGCTTTGGACCAAGTG
NDUFAB1 S112A Rev	AGGTCTTTCATAAAATGAGAATTTAC

**Table 4:** Aasdhppt genomic primers.

Primers for Genomic Amplification of *Aasdhppt*	

Forward primer	TACCTGGCTTGAGGATCAGC
Reverse primer	GGTTCTGCAGCAAGAACTGCAT

**Table 5:** Antibodies.

Antibodies	Source and reference number

AASDHPPT	Proteintech 11,244–1-AP
β-actin	Proteintech 66,009–1-Ig
NDUFA9	Abcam ab14713
UQCRFS1	Proteintech 18,443–1-AP
MTCO1	Abcam ab110270
ATP5a	Abcam ab14748
Lipoic acid	Millipore 437,695
Pyruvate Dehydrogenase E2 (DLAT)	Abcam ab172617
DLST	Cell Signaling 5556
SDHB	Abcam ab14714
NDUFAB1	Abcam ab96230 and ab181021
GFP	Abcam ab290
Citrate Synthase	Cell Signaling 14,309
Anti-V5	Abcam ab27671
Anti-FLAG	Thermo Fisher MA1-91878
MECR	Proteintech 51,027–2-AP
Mitofusin-2	Cell Signaling 9482
SMAC/DIABLO	Cell Signaling 15,108
Goat anti-Mouse IgG (H + L) AlexaFluor 568	Thermo Fisher A-11004
Goat anti-Mouse IgG (H + L) Alexafluor 488	Thermo Fisher A-11029
Goat anti-Rabbit IgG (H + L) AlexaFluor 568	Thermo Fisher A-11011
Goat anti-Mouse IgG (H + L), HRP	Thermo Fisher A28177
Goat anti-Rabbit IgG (H + L), HRP	Thermo Fisher A16110
Goat anti-Mouse IgG (H + L) Dylight 800	Rockland Immunochemicals 610–145–002-0.5
Goat anti-Rabbit IgG (H + L) Dylight 800	Rockland Immunochemicals 611–145–002-0.5
Goat anti-Mouse IgG (H + L) AlexaFluor 680	Thermo Fisher A-21057
Goat anti-Rabbit IgG (H + L) AlexaFluor 680	Thermo Fisher A10043

## Data Availability

All data needed to evaluate the conclusions in the manuscript are present in the manuscript and/or Supplementary Information. Additional data related to this paper may be requested from the authors.

## Abbreviations:

4’PP: 4′-phosphopantetheine/yl
AASDHPPT: aminoadipate semialdehyde dehydrogenase phosphopantetheinyl transferase
ALDH1L2: Aldehyde Dehydrogenase 1 Family Member L2
ATP5a: ATP synthase F1 subunit alpha
BiFC: bimolecular fluorescence complementation
CoA: coenzyme A
CS: citrate synthase
DHRS2: mitochondrial Dehydrogenase/Reductase 2
DLAT: dihydrolipoyllysine-residue acetyltransferase
DLST: dihydrolipoamide S-succinyltransferase
ETC: electron transport chain
FAS: fatty acid synthesis
FASN: fatty acid synthase
FeS: iron sulfur cluster
LA: lipoic acid
LCMS: liquid chromatography mass spectrometry
LYRM: leucine tyrosine arginine motif
mtFAS: mitochondrial fatty acid synthesis
MFN2: mitofusin 2
MTS: mitochondrial targeting sequence
NAD+: nicotinamide adenine dinucleotide (oxidized form)
NDUFAB1: NADH:ubiquinone oxidoreductase subunit AB1
OGDH: oxoglutarate dehydrogenase
PAGE: polyacrylamide gel electrophoresis
PDH: pyruvate dehydrogenase
PMS: post-mitochondrial supernatant
PPT: mammalian AASDHPPT
PPTase: phosphopantetheinyl transferase
PTM: posttranslational modification
SDHB: succinate dehydrogenase subunit B
TCA: tricarboxylic acid
VC: venus c’ terminus
VN: venus n’ terminus
VUS: variant of uncertain significance
