# Supplementary figures and images for: Mitochondrial phosphopantetheinylation is required for oxidative metabolism
Source: Metabolism. Author manuscript; Available in PMC 2025 Dec 2. (PMC12671336; doi:10.1016/j.metabol.2025.156413)

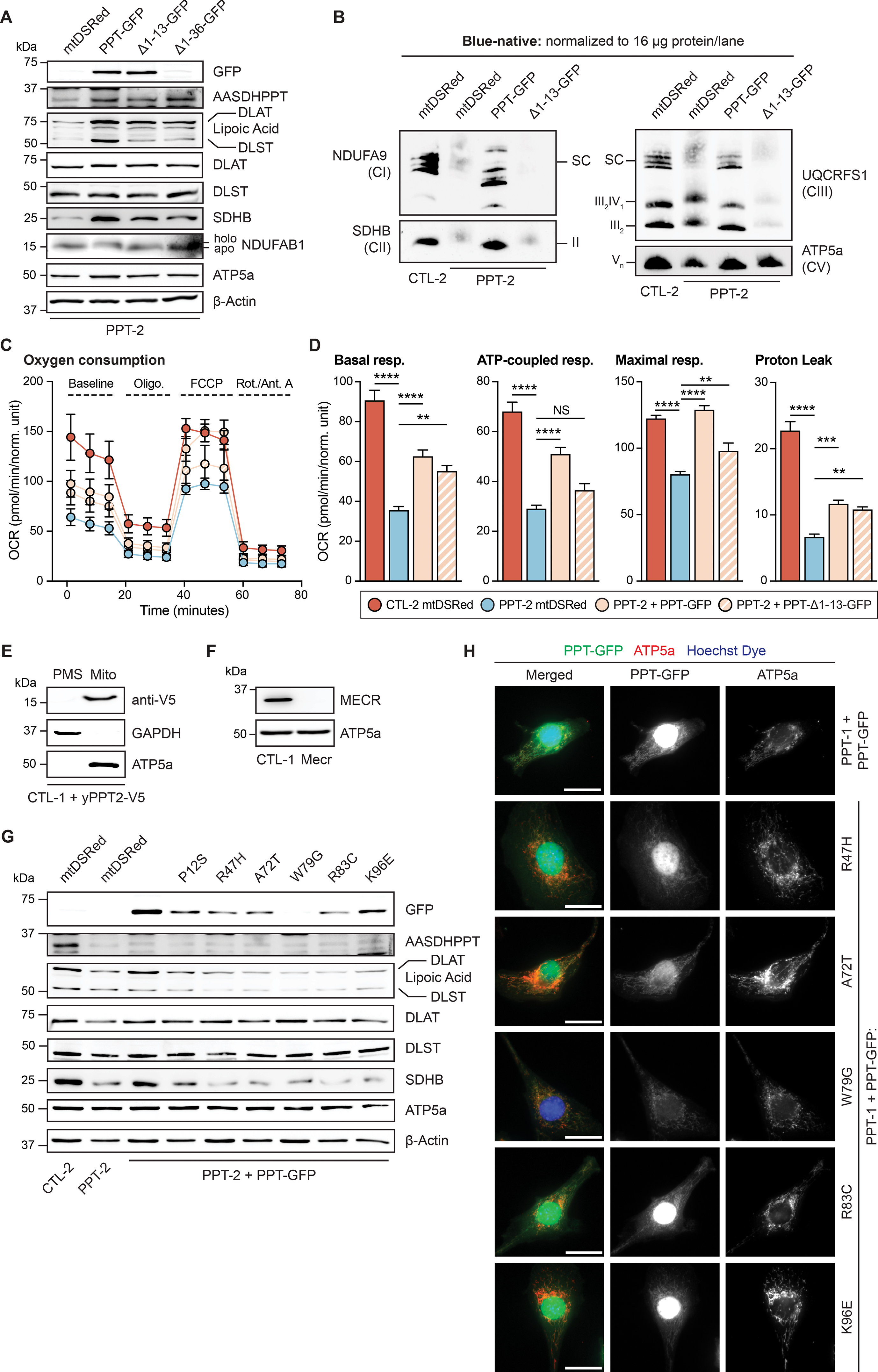

Supplement: MMC5 [file NIHMS2116987-supplement-MMC5.jpg]

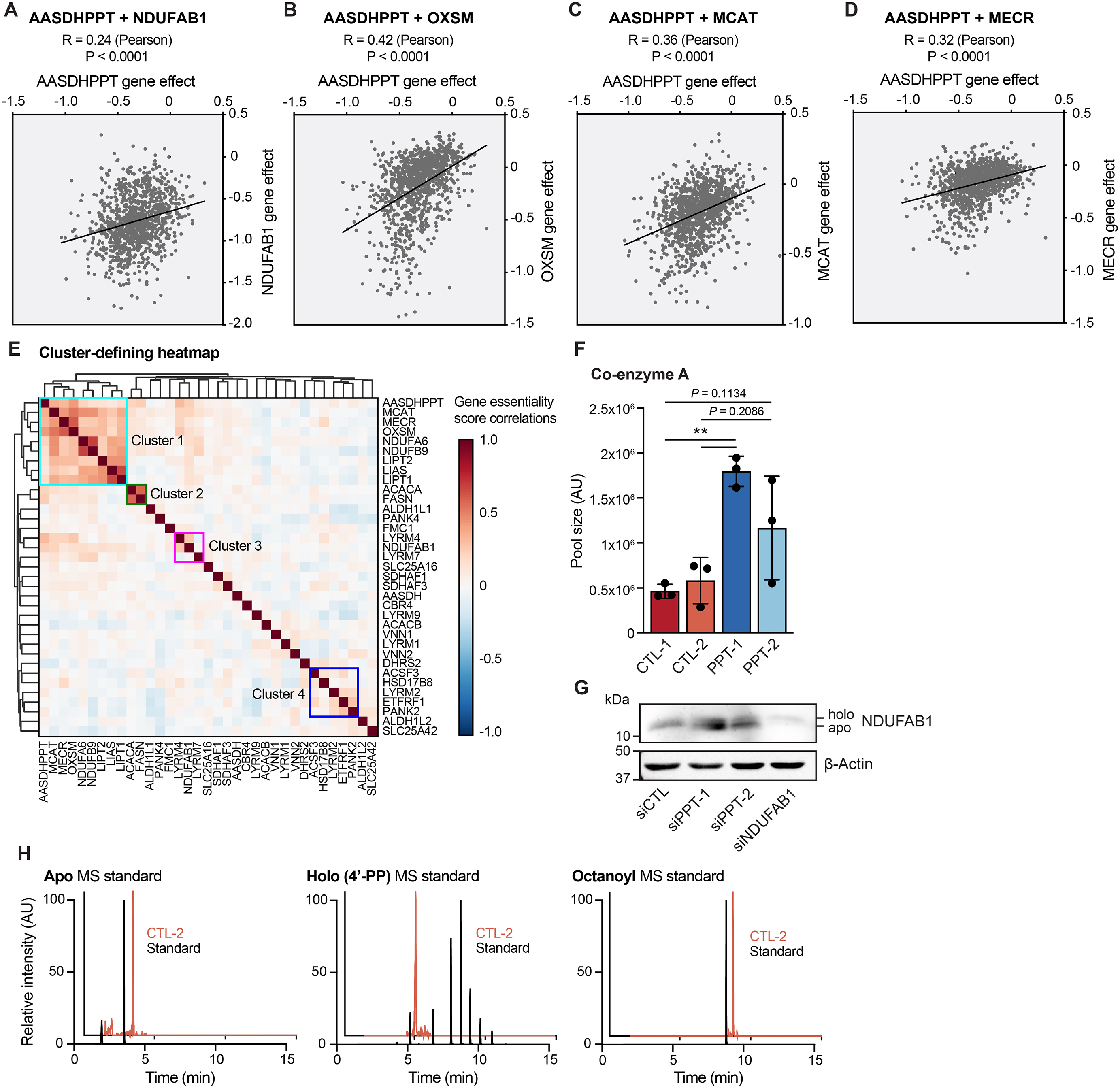

Supplement: MMC2 [file NIHMS2116987-supplement-MMC2.jpg]

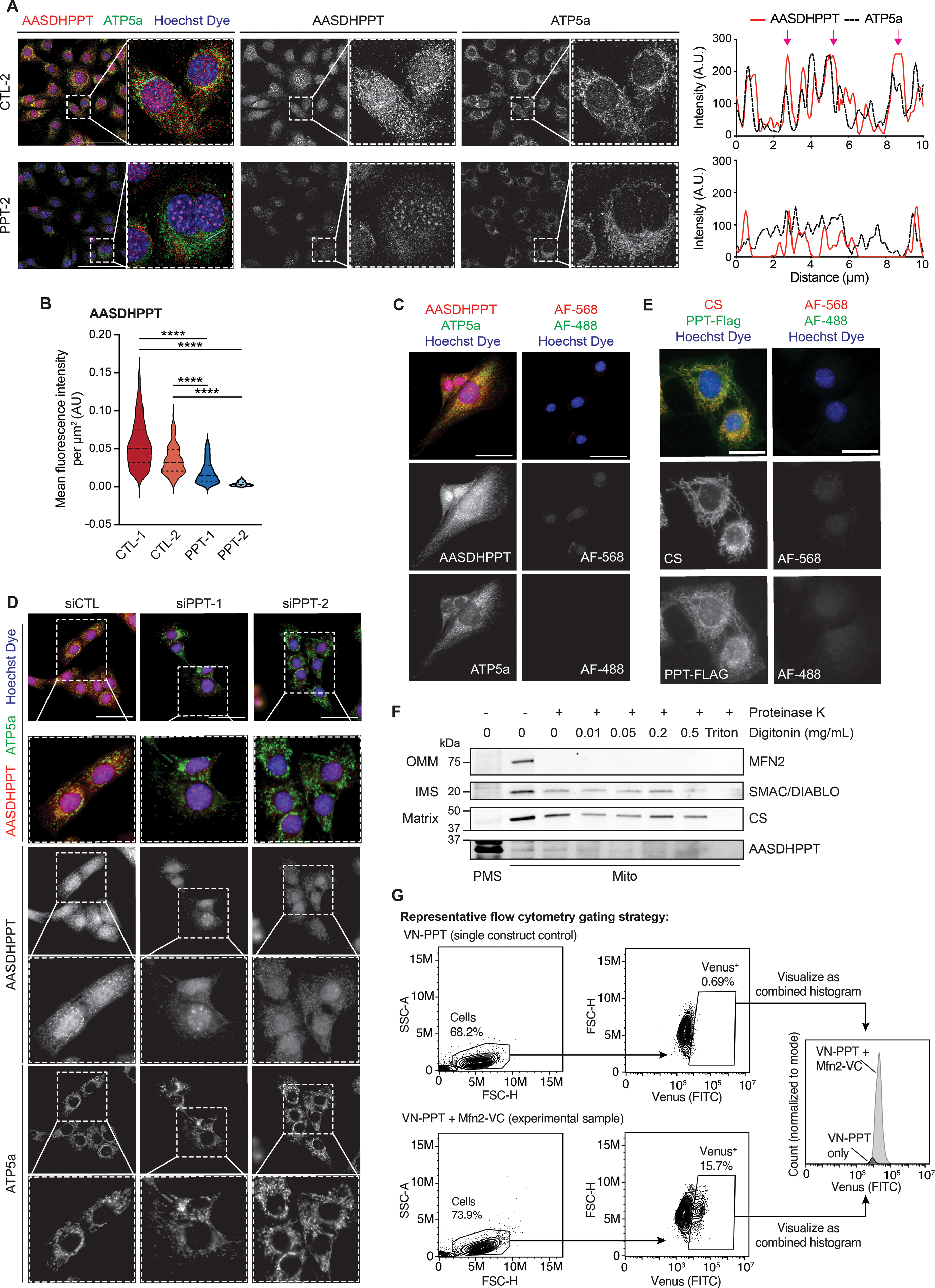

Supplement: MMC3 [file NIHMS2116987-supplement-MMC3.jpg]

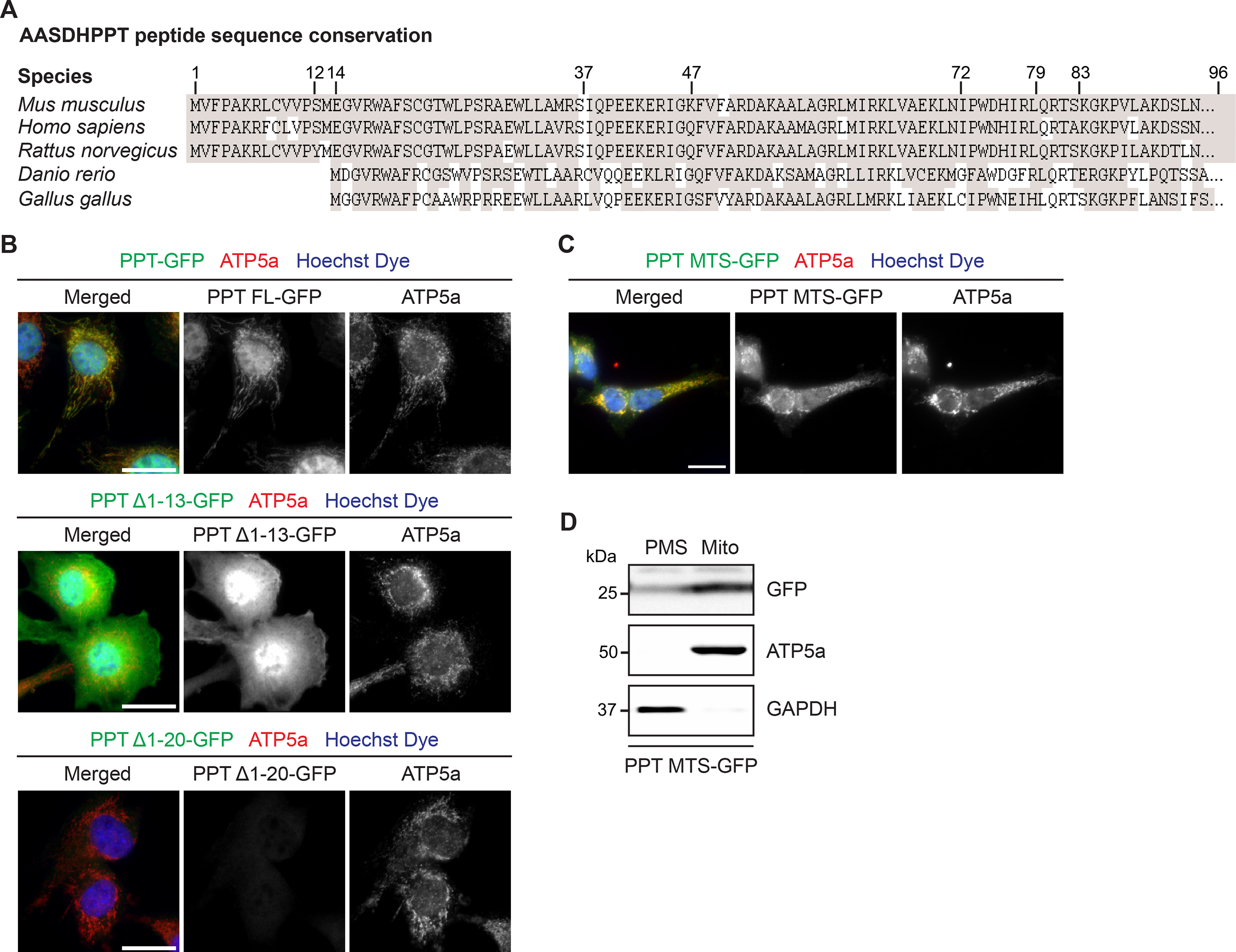

Supplement: MMC4 [file NIHMS2116987-supplement-MMC4.jpg]

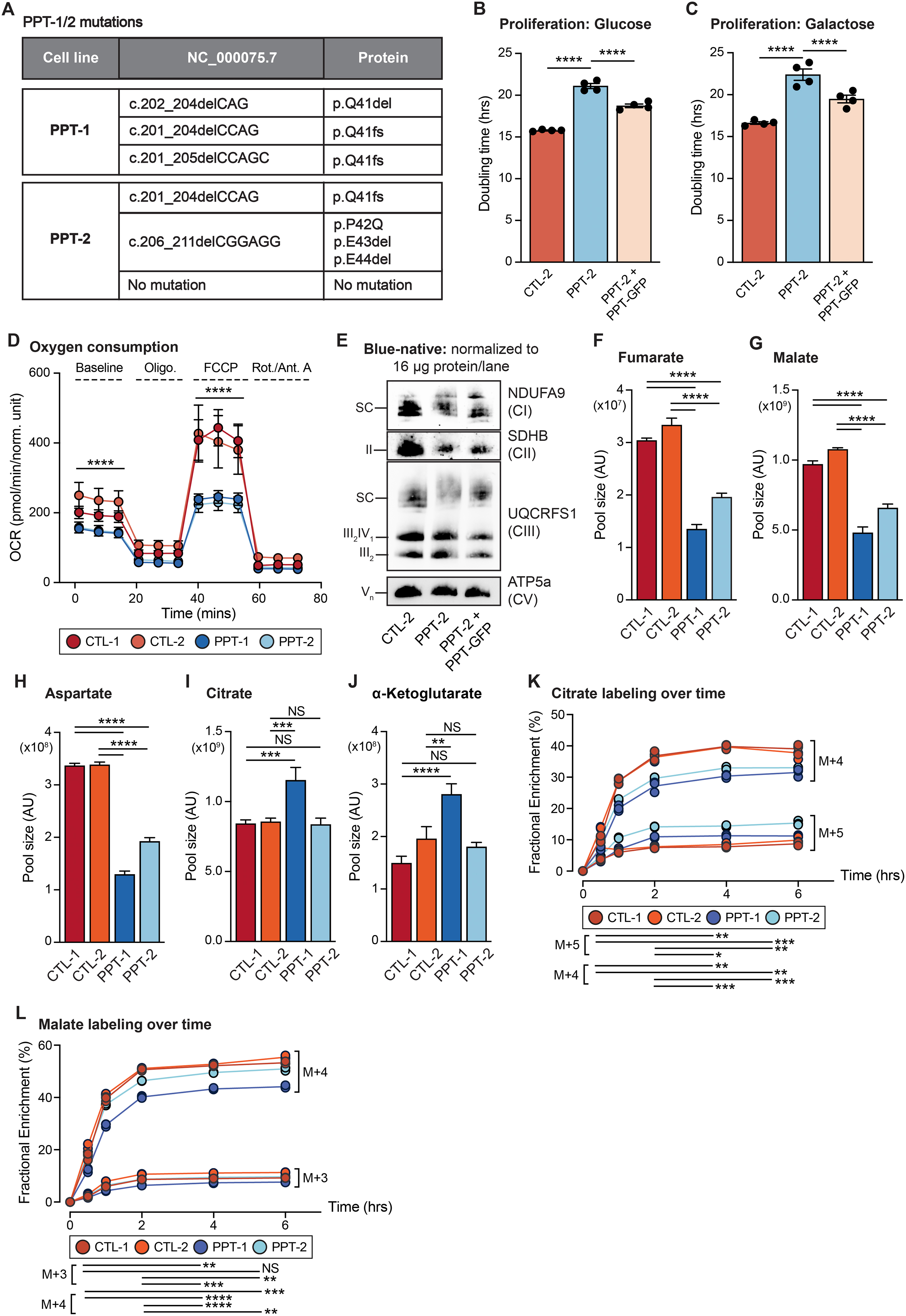

Supplement: MMC1 [file NIHMS2116987-supplement-MMC1.jpg]
